# Construction and validation of a risk prediction model for postoperative lung infection in elderly patients with lung cancer

**DOI:** 10.1097/MD.0000000000040337

**Published:** 2024-11-01

**Authors:** Xiaojie Chen, Lixin Wu, Gang Lan, Xiaofeng Li, Xuejing Wang, Ping Zhang, Weihu Huang

**Affiliations:** a Department of Pulmonary and Critical Care Medicine, Zhejiang Rongjun Hospital, Jiaxing City, Zhejiang Province, China; b Department of Chest Disease Diagnosis and Treatment Center, Zhejiang Rongjun Hospital, Jiaxing City, Zhejiang Province, China.

**Keywords:** elderly lung cancer, lung infection, predictive model, risk factors

## Abstract

This study aimed to analyze the risk factors for postoperative lung infection in elderly patients with lung cancer (LC) and construct a predictive model. A retrospective analysis was conducted on 192 elderly patients with LC who underwent surgical treatment in our hospital between February 2020 and May 2023. According to whether there is lung infection after surgery, they were divided into an infected group (n = 55) and a noninfected group (n = 137). Binary logistic regression was used to analyze factors influencing postoperative lung infection in elderly patients with LC. Based on the logistic regression results, a predictive model for postoperative lung infection in LC patients was constructed. The receiver operating characteristic curve was used to analyze C-reactive protein (CRP), interleukin-6 (IL-6), insulin-like growth factor-1 (IGF-1), and their combination in predicting postoperative lung infection in patients with LC. There were significant differences between the infected group and the noninfected group in age, smoking history, diabetes, and perioperative antibiotic use were significantly different between the infected and noninfected groups (*P* < .05). The postoperative CRP, IL-6, and IGF-1 levels in the infected group were higher than those in the noninfected group on the 1st day (*P* < .05). Logistic regression analysis showed that age > 70 years, history of smoking, history of diabetes, prolonged use of perioperative antibiotics, and elevated CRP, IL-6, and IGF-1 levels on the 1st day after surgery were risk factors for postoperative lung infection in elderly patients with LC (*P* < .05). Receiver operating characteristic curve analysis showed that the area under curve values of CRP, IL-6, IGF-1, and their combination in predicting postoperative lung infection in elderly patients with LC were 0.701, 0.806, 0.737, and 0.871, *P* < .05), with sensitivity values of 0.443, 0.987, 0.456, and 0.835, respectively; the specificity was 0.978, 0.525, 0.991, and 0.821, respectively. Age > 70 years, smoking history, diabetes history, prolonged use of perioperative antibiotics, and elevated CRP, IL-6, and IGF-1 levels on the 1st day after surgery have an impact on postoperative lung infection in elderly patients with LC. Early postoperative monitoring of changes in CRP, IL-6, and IGF-1 levels can provide an important reference for predicting the occurrence of postoperative lung infections.

## 1. Introduction

Lung cancer (LC) is a common malignant tumor in clinical practice. In clinical practice, surgical treatment, such as pneumonectomy, lobectomy, and segmental/wedge-shaped resection, is often chosen for this disease. Controlling the progression of the disease and improving prognosis by removing the diseased lung.^[1]^ However, through clinical investigations, it has been found that LC patients who undergo surgical treatment are highly susceptible to postoperative lung infection.^[2]^ According to literature reports, the incidence of postoperative infection in LC is approximately 5.30% to 22.00%, and this infection can lead to acute respiratory distress syndrome and even death.^[3]^ Therefore, studying the relevant factors that affect postoperative lung infection in patients with LC and finding effective models to predict postoperative lung infection are of great significance for improving the treatment effectiveness and quality of life of patients. C-reactive protein (CRP) is an important indicator for clinical observation of the inflammatory response. Under normal physiological conditions, its content is extremely low, but under the influence of inflammatory factors, it can significantly increase the expression level of CRP.^[4]^ Interleukin-6 (IL-6) is a pro-inflammatory cytokine that is upregulated by the invasion of pathogenic bacteria and participates in the inflammatory response through synergistic effects with various cytokines.^[5]^ Insulin-like growth factor-1 (IGF-1) is a peptide-growth factor. According to previous studies, inflammation can promote changes in the body’s IGF-1 level, inducing lymphocyte differentiation and immunoglobulin production by increasing its level and inhibiting inflammation.^[6]^ However, the specific mechanisms of CRP, IL-6, and IGF-1 in postoperative secondary infections in LC patients need to be further clarified. Therefore, this study aimed to explore the risk factors for postoperative lung infection in elderly patients with LC and construct predictive models based on PCRP, IL-6, and IGF-1 to provide a reference for the diagnosis and treatment of postoperative lung infections in elderly patients with LC.

## 2. Materials and methods

### 2.1. Patients

We retrospectively collected clinical data from 192 elderly patients with LC who underwent surgical treatment at our hospital from February 2020 to May 2023. They were divided into an infected group (n = 55) and a noninfected group (n = 137) based on whether there was lung infection after surgery.

All procedures conducted in research involving human participants complied with the ethical standards of institutions and/or national research committees, as well as the Helsinki Declaration (revised in 2013). The informed consent was waived by the ethics committee for the observational and retrospective nature. Zhejiang Rongjun Hospital medical ethics committee approved this study (No. RL20220813).

### Inclusion criteria:

- Patients diagnosed with LC through pathological examination;
- Tumor, node, and metastasis (TNM) staging was from stage I to II;
- Patients with complete clinical data.

### Exclusion criteria:

- Patients with other malignant tumors;
- Patients with mental disorders;
- Patients with systemic infectious diseases.

*Diagnostic criteria for postoperative lung infection*. (1) Main manifestations: (a) appearance of thick phlegm or changes in the nature of phlegm; (b) increased secretion from the respiratory system or the need for sputum aspiration; (c) having cough, difficulty breathing, or shortness of breath; (d) worsening of ventilation disorders; (2) Secondary manifestations: (a) body temperature > 38 °C; (b) white blood cell count < 4 × 10^9^/L or white blood cell count > 12 × 10^9^/L. The diagnosis can be made by meeting any two of the main manifestations and any one of the secondary manifestations.

Sex, BMI, pathological type, surgical method, hypertension, maximum tumor diameter, TNM stage, age, smoking history, forced expiratory volume in 1 second/forced vital capacity ratio, diabetes, perioperative antibiotic use time, and CRP, IL-6, and IGF-1 levels on the 1st day after surgery were compared between the 2 groups. Binary logistic regression was used to analyze factors influencing postoperative lung infection in elderly patients with LC. Based on the logistic regression results, a combined model of CRP, IL-6, and IGF-1 levels was constructed to predict postoperative lung infection in patients with LC. Receiver operating characteristic (ROC) curves were drawn to analyze CRP, IL-6, IGF-1, and their combination in predicting postoperative lung infection in patients with LC.

### 2.2. Statistical analysis

SPSS22.0 software was used to analyze the data. The continuous data that conformed to the normal distribution were represented by ($\bar{x}\pm s$) and subjected to an independent sample *t* test. Categorical variables were represented by (n, %), and compared using the chi-square test. The influencing factors were analyzed using a binary logistic regression model. *P* < .05 was considered statistically significant.

## 3. Results

**Figure 1.**
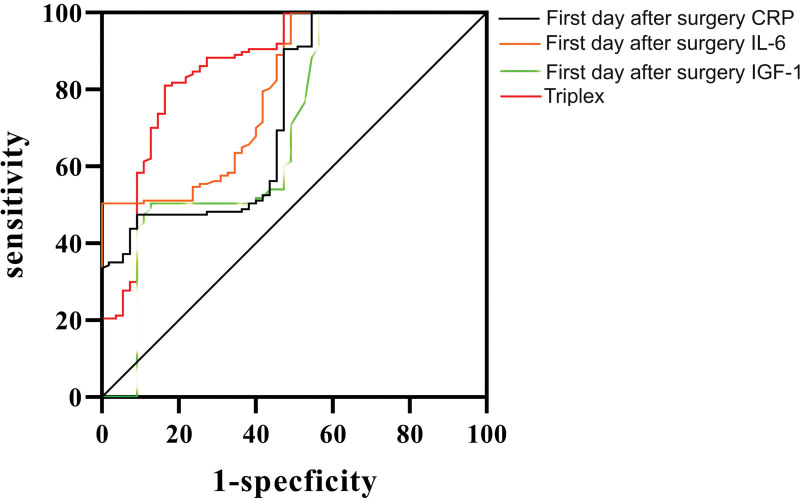
Analysis of the predictive efficacy of postoperative 1dCRP, IL-6, IGF-1, and IGF-3 combined methods in predicting secondary lung infection in elderly patients after lung cancer surgery. CRP = C-reactive protein, IGF-1 = insulin-like growth factor-1, IGF-3 = insulin-like growth factor-3, IL-6 = interleukin-6.

There were no statistically significant differences between the 2 groups in terms of sex, BMI, pathological type, surgical method, hypertension, maximum tumor diameter, and TNM staging (*P* > .05). However, in the comparison of age, smoking history, diabetes, and perioperative antibiotic use time, the difference was statistically significant (*P* < .05) (Table 1). The CRP, IL-6, and IGF-1 levels in the infected group were higher than those in the noninfected group on the 1st day after surgery (*P* < .05) (Table 2). The binary logistic regression model showed that age > 70 years; smoking history; diabetes history; prolonged use of perioperative antibiotics; and elevated CRP, IL-6, and IGF-1 levels on the 1st day after surgery were risk factors for postoperative lung infection in elderly patients with LC (*P* < .05) (Table 3). ROC curves were established to analyze CRP, IL-6, and IGF-1 levels and their combined prediction of postoperative lung infection in elderly patients with LC. The area under curve (AUC) values of CRP, IL-6, IGF-1, and their combination in predicting postoperative lung infection in elderly patients with LC were 0.701, 0.806, 0.737, and 0.871, *P* < .05), with sensitivity values of 0.443, 0.987, 0.456, and 0.835, respectively; The specificity was 0.978, 0.525, 0.991, and 0.821, respectively (Table 4 and Fig. 1).

**Table 1:** Comparison of baseline data between the 2 groups.

Baseline data		Infected group (n = 55)	Noninfected group (n = 137)	*χ*^2^/*t*	*P*
Gender	Male	34 (61.82)	72 (52.55)	1.362	.243
	Female	21 (38.18)	65 (47.45)		
Age	>70 years old	31 (56.36)	51 (37.23)	5.874	.015
	≤70 years old	24 (43.64)	86 (62.77)		
History of smoking	Yes	34 (61.82)	60 (43.80)	5.101	.24
	No	21 (38.18)	77 (56.20)		
BMI	>23 kg/m^2^	28 (50.91)	69 (50.36)	0.005	.946
	≤23 kg/m^2^	27 (49.09)	68 (49.64)		
Pathological type	Small cell lung cancer	29 (52.73)	75 (54.74)	0.064	.800
	Non-small cell lung cancer	26 (47.27)	62 (45.26)		
Mode of operation	Total lobectomy	21 (38.18)	56 (40.88)	0.127	.939
	Lobectomy	19 (34.55)	46 (33.58)		
	Segmental/wedge resection	15 (27.27)	35 (25.55)		
History of diabetes	Yes	24 (43.64)	34 (24.82)	6.592	.010
	No	31 (56.36)	103 (75.18)		
Hypertension	Yes	26 (47.27)	45 (32.85)	3.505	.061
	No	29 (52.73)	92 (67.15)		
Maximum tumor diameter	<22 mm	37 (67.27)	105 (76.64)	1.789	.181
	≥22 mm	18 (32.73)	32 (23.36)		
TNM staging	Stage I	21 (38.18)	36 (26.28)	2.664	.103
	Stage II	34 (61.82)	101 (73.72)		
Perioperative antibiotic use time		8.35 ± 1.47	7.12 ± 1.22	5.724	<.001

TNM = tumor, node, and metastasis.

**Table 2:** Comparison of serum CRP, IL-6, and IGF-1 1st day after surgery between the 2 groups.

Group	First day after surgery CRP (mg/L)	First day after surgery IL-6 (pg/mL)	First day after surgery IGF-1 (ng/mL)
Infected group (n = 55)	9.52 ± 3.69	125.62 ± 34.96	242.32 ± 62.78
Noninfected group (n = 137)	7.11 ± 2.14	75.24 ± 22.96	188.45 ± 48.63
*t*	5.651	11.725	6.363
*P*	<.001	<.001	<.001

AUC = area under curve, CRP = C-reactive protein, IGF-1 = insulin-like growth factor-1, IL-6 = interleukin-6.

**Table 3:** Binary logistic regression analysis affecting secondary lung infection in elderly patients with lung cancer after surgery.

Variable	B	SE	Wald	*P*	OR (95% CI)
Age	0.818	0.336	5.930	.015	2.265 (1.173–4.375)
History of smoking	0.741	0.338	4.819	.028	2.099 (1.083–4.069)
History of diabetes	0.864	0.349	6.143	.013	2.372 (1.198–4.697)
Perioperative antibiotic use time	0.645	0.132	23.958	<.001	1.906 (1.472–2.468)
First day after surgery CRP	0.321	0.068	21.970	<.001	1.378 (1.205–1.576)
First day after surgery IL-6	0.063	0.011	33.913	<.001	1.064 (1.042–1.087)
First day after surgery IGF-1	0.018	0.003	27.997	<.001	1.018 (1.011–1.025)

AUC = area under curve, CRP = C-reactive protein, IGF-1 = insulin-like growth factor-1, IL-6 = interleukin-6.

**Table 4:** Analysis of the predictive efficacy of postoperative 1dCRP, IL-6, IGF-1, and IGF-3 combined methods in predicting secondary lung infection in elderly patients after lung cancer surgery.

Variable	AUC	SE	*P*	95% CI	Cutoff	Jorden index	Sensitivity	Specificity
First day after surgery CRP	0.698	0.046	.000	0.608–0.789	9.850	0.421	0.443	0.978
First day after surgery IL-6	0.801	0.035	.000	0.733–0.869	105.25	0.502	0.509	0.993
First day after surgery IGF-1	0.744	0.040	.000	0.665–0.823	263.15	0.448	0.455	0.993
Triplex	0.867	0.032	.000	0.804–0.930	37.901	0.646	0.836	0.810

AUC = area under curve, CRP = C-reactive protein, IGF-1 = insulin-like growth factor-1, IGF-3 = insulin-like growth factor-3, IL-6 = interleukin-6.

## 4. Discussion

The study showed that age > 70 years, history of smoking , history of diabetes, prolonged use of perioperative antibiotics, and elevated CRP, IL-6, and IGF-1 levels on the 1st day after surgery were risk factors for postoperative lung infection in elderly LC patients. Based on the age analysis, it was found that the mechanism undergoes degenerative changes with increasing age. Especially for LC patients over 70 years old, their body resistance, ciliary motility of the tracheobronchial mucosa, respiratory defense function, cough response, and elastic function of lung tissue will all be weakened to varying degrees, which increases the risk of postoperative lung infection by affecting the patient’s sputum excretion ability.^[7]^ In addition, residual anesthetic drugs in the short term after surgery may inhibit respiratory muscle function and weaken the cough reflex; even if the anesthesia completely subsides, normal breathing movements can be affected by factors such as wound pain and abdominal bloating. In addition, people over 70 years of age have weaker physical fitness than those under 70 years of age. Postoperative bed rest time will also be longer, causing some secretions in the lung tissue to accumulate, leading to sputum coughing and lung infections.^[4]^ Xu et al^[8]^ found that smoking can damage lung health. The reason for this may be that nicotine in tobacco reduces the resistance of the lungs to external pathogens through the structure and function of lung tissue; At the same time, smoking can also increase the risk of postoperative lung infection by inhibiting the movement of respiratory mucosal cilia and weakening the function of bronchoalveolar macrophages, making it difficult to cough up sputum.^[9]^ Analysis of postoperative lung infections in elderly patients with LC with a history of diabetes prolonged exposure to high blood sugar can easily lead to a weakened immune system. If chemotaxis of white blood cells is reduced, their phagocytosis and lethality are weakened, and the body’s defense against pathogens is weakened, the risk of postoperative lung infection is increased.^[10]^ In addition, patients with a history of diabetes often have vascular diseases such as pulmonary microvascular diseases, which are prone to pulmonary hypoxia and oxygen diffusion dysfunction. These pathological changes can provide favorable conditions for the reproduction of pathogenic bacteria, thereby increasing the risk of lung infection.^[11]^ Studies have also shown that microcirculation disorders caused by diabetes can delay the healing of surgical wounds and increase the risk of infection.^[12]^ The purpose of using antibiotics during the perioperative period is to prevent postoperative infections and kill or inhibit cell growth and reproduction. However, clinical research has found that the longer the use of antibiotics, the better the prevention of postoperative infections. However, the longer the use of antibiotics, the more likely it is to lead to an imbalance in the microbiota of the body.^[13]^ Antibiotics not only have antibacterial effects but also regulate immunity. Proper use of antibiotics during the perioperative period can enhance resistance to pathogens. However, prolonged use of antibiotics can cause adverse reactions, such as immune system suppression, which reduces the body’s resistance to pathogens and provides favorable conditions for pathogens to invade the lung tissue.^[14]^ Therefore, it is believed that when using antibiotics during the perioperative period, it is necessary to determine the usage time based on the specific situation of the patient, in order to prevent postoperative infection and avoid adverse effects on the body; At the same time, it is also necessary to evaluate the patient’s physical condition before using antibiotics, and combine with common pathogens and susceptibility factors of postoperative lung infections in the past to choose appropriate antibiotics, rather than blindly using high-limit antibiotics.

CRP is an acute phase response protein that recognizes and binds pathogens, such as bacteria, fungi, and parasites, activates complement, and increases monocyte phagocytosis of pathogens.^[15]^ At the same time, it also participates in the immune regulation of the body, promoting the Th1 cell immune response, leading to inflammation regression and tissue repair. According to the analysis of changes in CRP levels, when the body experiences infection, inflammation, or tissue damage, its expression shows a significant upward trend.^[16]^ According to clinical research, it has been found that the upregulation of CRP expression levels in the early postoperative period, that is, 1 to 3 days after surgery, may be related to acute inflammatory response caused by surgical trauma. This study analyzed the CRP levels of LC patients on the 1st day after surgery and found that LC patients with postoperative lung infection had significantly higher CRP levels than those without postoperative lung infection. From this, it can be seen that early monitoring of changes in CRP levels after surgery is beneficial for providing important reference basis for evaluating the occurrence of lung infection. IL-6 is a multifunctional cytokine that participates in various biological processes such as immune responses, inflammatory responses, and tissue repair. Based on the analysis of its mechanism of action in immune response, it was found that IL-6 can promote antibody production by stimulating the proliferation and differentiation of T and B cells, and by regulating the humoral immune response, the body’s defense against pathogenic bacteria is enhanced. In the inflammatory response, IL-6 can aggravate the inflammatory response by stimulating the release of large amounts of inflammatory factors such as IL-1 and IL-8. In patients with postoperative lung infection, elevated IL-6 levels can further damage and destroy the lung tissue by exacerbating the inflammatory response. IL-6 promotes wound healing and tissue repair by stimulating proliferation and differentiation of fibroblasts and keratinocytes.^[17]^ The results of this study showed that IL-6 levels in the infected group were significantly higher than those in the noninfected group, indicating that IL-6 is involved in the postoperative lung infection process. IGF-1 is a peptide substance and its content is relatively low under normal circumstances. Once lung infection occurs, IGF-1 levels will significantly increase to inhibit the release of inflammatory factors and enhance the body’s defense ability by stimulating immune cell proliferation and differentiation.^[18]^ Studies also indicating that an increase in IGF-1 levels may mediate infiltration of macrophages and monocytes in lung tissue, exacerbating inflammatory damage.^[19]^ In the comparison of IGF-1 levels between the infected and noninfected groups in this study, it was found that the level of IGF-1 in the Infected group was higher, indicating that monitoring the expression level of IGF-1 can provide guidance for the diagnosis and treatment of postoperative lung infections. This study is also based on this point and believes that the above indicators have a certain value in the diagnosis of postoperative lung infection in patients with LC. Incorporating CRP, IL-6, and IGF-1 into the ROC curve model, the results showed that the AUC values of CRP, IL-6, and IGF-1 for predicting postoperative secondary infections in patients with LC were 0.701, 0.806, and 0.737, respectively, further confirming the reliability of the above speculation. However, further research in this study found that the combined prediction efficiency of CRP, IL-6, and IGF-1 was higher, with the AUC value (0.871) of the 3 combinations being significantly higher than the detection of CRP, IL-6, and IGF-1 alone. Different biomarkers may reflect different characteristics of postoperative lung infection, and combining them is beneficial for a more comprehensive assessment of the patient’s lung infection situation.

Limitations of this study: The sample size was relatively small, and all patients came from the same hospital, which may have some bias. Large-sample, multicenter studies are needed to further demonstrate the reliability of the conclusions.

## 5. Conclusion

Age > 70 years, smoking history, diabetes history, prolonged use of perioperative antibiotics, and elevated CRP, IL-6, and IGF-1 levels on the 1st day after surgery have an impact on postoperative lung infection in elderly LC patients. Early monitoring of changes in CRP, IL-6, and IGF-1 levels after surgery can provide important reference values for predicting the occurrence of postoperative lung infections.

## Author contributions

**Conceptualization:** Xiaojie Chen.

**Formal analysis:** Xiaofeng Li.

**Investigation:** Gang Lan, Ping Zhang.

**Methodology:** Lixin Wu.

**Software:** Xuejing Wang.

**Writing – original draft:** Xiaojie Chen.

**Writing – review & editing:** Weihu Huang.
